# Fragmentation Impairs the Microclimate Buffering Effect of Tropical Forests

**DOI:** 10.1371/journal.pone.0058093

**Published:** 2013-03-04

**Authors:** Robert M. Ewers, Cristina Banks-Leite

**Affiliations:** Imperial College London, Silwood Park Campus, Ascot, United KIngdom; The Ohio State University, United States of America

## Abstract

**Background:**

Tropical forest species are among the most sensitive to changing climatic conditions, and the forest they inhabit helps to buffer their microclimate from the variable climatic conditions outside the forest. However, habitat fragmentation and edge effects exposes vegetation to outside microclimatic conditions, thereby reducing the ability of the forest to buffer climatic variation. In this paper, we ask what proportion of forest in a fragmented ecosystem is impacted by altered microclimate conditions driven by edge effects, and extrapolate these results to the whole Atlantic Forest biome, one of the most disturbed biodiversity hotspots. To address these questions, we collected above and below ground temperature for a full year using temperature sensors placed in forest fragments of different sizes, and at different distances from the forest edge.

**Principal Findings:**

In the Atlantic forests of Brazil, we found that the buffering effect of forests reduced maximum outside temperatures by one third or more at ground level within a forest, with the buffering effect being stronger below-ground than one metre above-ground. The temperature buffering effect of forests was, however, reduced near forest edges with the edge effect extending up to 20 m inside the forest. The heavily fragmented nature of the Brazilian Atlantic forest means that 12% of the remaining biome experiences altered microclimate conditions.

**Conclusions:**

Our results add further information about the extent of edge effects in the Atlantic Forest, and we suggest that maintaining a low perimeter-to-area ratio may be a judicious method for minimizing the amount of forest area that experiences altered microclimatic conditions in this ecosystem.

## Introduction

Microclimate exerts considerable influence over the functioning of forest ecosystems [1], with direct influences on processes as diverse as soil respiration, nutrient cycling, plant regeneration and invertebrate mortality rates [2], [3]. Within forests, microclimate conditions are buffered from the macroclimatic conditions immediately adjacent to and above forests, having lower annual and seasonal variability reflected in warmer minimum temperatures and cooler maximum temperatures [4]. Forest fragmentation, and the creation of forest edges, exposes parts of the forest environment to external climatic conditions, reducing the ability of a forest to buffer its internal microclimate from those more extreme macroclimate conditions.

Altered microclimate conditions near forest edges are routinely reported from forests around the world. Air temperature gradients typically extend around 50–100 m inside a forest [4], [5], [6], [7], but the gradient itself can reverse with time of day, with temperature declining towards the forest interior during the day but increasing towards the interior during the night [5]. Humidity during the day is typically lower near forest edges [5], [6] and both the amount of light penetrating to the forest floor and wind speed drop dramatically with distance inside a forest [5], [8]. These changes to microclimate near forest edges have repeatedly been hypothesized to underlie the response of many invertebrate [9], plant [10] and bryophyte [11] species to forest edges [12]. Most reported edge effects on biodiversity tend to be restricted to relatively narrow bands around forest edges, but they can also extend over distances of more than one kilometre [13]. While there are numerous studies reporting air temperature gradients across forest edges [1], [12], there are far fewer studies that have examined soil temperature or temperature at ground level and in the litter layer [2]. Temperatures in these two strata are important as they potentially impact the composition of microbial and leaf litter invertebrate communities, both of which play important functional roles in forest ecosystems [2], [14].

When edge effects penetrate just short distances inside a forest, the temptation is to conclude that their impact on biodiversity is highly localized. This can, however, be a misleading conclusion because it is the spatial accumulation of edge effects integrated across all edges within a landscape that determines the total impact of edges in fragmented landscapes [15], [16], [17]. In fragmented landscapes such as the Atlantic Forest, most remnant forest patches are small (<100 ha) [18], [19] and consequently have high perimeter-to-area ratios [20]. It is likely, therefore, that the accumulation of even small-scale edge effects may impact large proportions of the standing forest in fragmented landscapes.

Our goal in this study was to estimate the proportion of forest cover in a fragmented ecosystem that is impacted by altered microclimate conditions near forest edges. We collected empirical data to quantify the extent to which tropical Atlantic forest buffers its forest interior microclimate from variation in the external macroclimate, and the spatial scale over which the buffering effect breaks down near forest edges. To address these questions, we used nearly 100 sensors that measured temperature every three hours over the course of a whole year, which, to our knowledge, makes this the longest running and most detailed data collection of edge induced microclimatic changes in the Neotropics. Field data were then combined with maps of Brazilian Atlantic forest remnants to determine the extent of microclimatic changes at biome scale. The Brazilian Atlantic forest is one of the world’s most imperiled biodiversity hotspots [21], with natural forest cover reduced in extent by 88% and the remainder heavily fragmented [19].

## Methods

### Field Data

**Figure 1:**
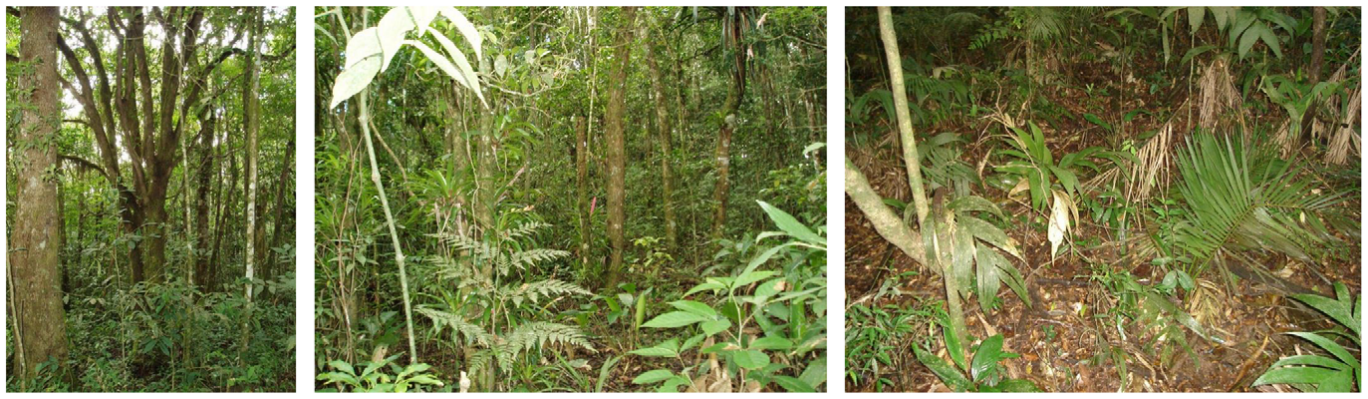
Photographs showing the typical structure of the (a) forest, (b) understory and (c) litter layer within the study area.

We conducted our study in the heavily fragmented Atlantic forest biome, working in a 10,000 ha landscape with 49% forest cover, which has been the site of a large study into the effects of forest fragmentation on bird and mammal communities (23°50′ S, 47°20′ W) [22], [23], [24], [25]. The present distribution of forest in this landscape is a result of a highly dynamic process of deforestation and regrowth over the past centuries [26]. Remnants are therefore a patchwork of vegetation of different successional stages, varying from intermediate to old secondary forest (Fig. 1). Because of this, vegetation structure at edges does not differ from that of forest interiors with respect to density of trees, size of trees and vegetation stratification [22]. Also, because fragments were comprised of secondary forest, there were few emergent trees and treefall gaps, meaning that the canopy was lower (canopy height ∼ 15 m) and much more homogeneous than old growth or primary forests (canopy height 20–35 m). Across 23 sites within the study area, we estimated understory foliage density in patch interiors by recording the number of points at which foliage touched a 2 m vertical pole [22]. Understory vegetation touched the pole an average of 23.3 (SE = 0.7) times within one metre of the ground, and an additional 15.4 (SE = 1.0) locations between 1 and 2 metres above ground, and there were no significant differences in the foliage density profile between edge and interior sites [22].

Within this landscape we selected eight forest fragments ranging in size from 4 to 1650 ha. We established one transect in each fragment, extending from the forest edge to the interior. Temperature data were collected on a log_4_ scale along this transect, with sites located at 1, 4, 16 and 64 m inside the forest. A log scale was used on the assumption that microclimate variables change most rapidly in close proximity to forest edges [4], and the maximum distance reflects the fact that most edge effects on temperature have been reported as penetrating <50 m into forest [12], [27]. However, microclimate conditions within forest are not only impacted by increasing distance from forest edges [4], but are also very different above and below-ground [28]. To determine if edge effects on temperature were more or less pronounced above or below-ground, one temperature sensor was suspended one metre above ground in the understory vegetation, another was placed in the leaf litter at ground level, and the third was buried 15 cm below ground. A further eight temperature sensors were placed in two sites outside the forest, suspended one metre above ground in the middle of pastures and separated by the same series of distances as temperature sensors on the forest edge transects. These external temperature sensors were more than 80 m from the nearest forest edge. Sensors located in the leaf litter and below ground were shielded by their matrix environment, and we did not provide artificial shields for temperature sensors located above ground. The combination of eight fragments, four edge distances, three height strata plus eight external sites gave a total of 104 temperature sensors, of which we were able to retrieve 73. The remaining 31 temperature sensors were lost due to animals digging them up and/or moving them, or to human interference including theft. To ensure that the large number of missing temperature sensors did not introduce any bias into our analyses of spatial patterns of microclimate, we conducted a binomial general linear model that found no significant effects of distance to edge (*z* = 0.01, *P* = 0.99), height strata (*z* = 0.50, *P* = 0.62) or their interaction (*z* = −0.01, *P* = 0.99) on the presence or absence of temperature sensors. Consequently, the spatial pattern of temperature sensor loss did not introduce any bias into our spatial analyses.

All sites were located within a tightly constrained altitudinal band (range 880–980 m.a.s.l.) to minimize the effect of this potentially confounding variable on our results. To ensure this 100-m altitudinal variation did not influence our models or conclusions, altitude was added as an offset to all statistical models. Temperature sensors were synchronized and temperature recorded every three hours for the 12 month period 1 September 2009 to 31 August 2010. We used iButton DS1922L-F5 dataloggers (Dallas Semiconductor), accurate to ±0.5°C between the temperature range of −10 to +65°C.

No specific permits were required for the described field studies, as we only collected data on temperature. The study was conducted in private land, and permission to enter the property was given by individual land owners.

### Buffering Effect

To quantify the average microclimate buffering effect of forest, we compared the temperature observed within the forest to the external air temperature. For each temperature sensor inside the forest, we had 365 days×8 measurements per day = 2920 paired observations of temperature recorded by that sensor and temperature recorded at the same time in the external environment. We regressed forest temperature at the sensor against external temperature and recorded the slope of the line as a measure of the buffering effect. This regression was conducted separately for each temperature sensor, thereby estimating a unique value of the buffering effect for each of the fragment×distance to edge×height strata locations.

We also estimated the buffering effect on daily maximum and minimum temperatures. Here, we recorded the maximum and minimum temperature recorded by each temperature sensor, giving 365 paired observations of extreme temperatures recorded by that sensor and extreme temperatures recorded on the same day in the external environment. As above, we regressed extreme temperature in the forest against extreme temperature in the external environment, and took the slope of the line as a measure of the buffering effect on extreme temperatures at each of the fragment×distance to edge×height strata locations.

Buffering effect values of one indicate no buffering (forest temperature is the same as external temperature), values less than one represent cooling effects of forest (forest temperature is lower than external temperature) and values greater than one would represent heating effects (forest temperature is higher than external temperature). Because the external temperature sensors were unshaded, the absolute estimates of buffering effects that we present almost certainly overestimate the actual buffering effect. However, because all temperatures recorded from sensors inside forest were compared to the same sensors recording temperature outside forest, the relative pattern of microclimate buffering that we report is robust.

### Statistical Analysis

To determine which, if any, of our three measures of buffering effect were strongest, we compared the strength of the average, daily maximum and daily minimum buffering effects using paired *t*-tests. We used analysis of covariance (ANCOVA) to test for the effects of distance from forest edge, height strata and their interaction on the buffering effect of forest. We conducted separate ANCOVA models to determine the impacts of these spatial variables on the average buffering effect, daily maximum and daily minimum buffering effects. In all analyses, distance from edge was log_4_-transformed and modeled as a continuous variable, whereas height strata was modeled as a categorical variable.

We tested for a seasonal effect on the average buffering effect of forest by estimating buffering effect at each location separately for the three hottest (December – February) and three coldest (June – August) months of the year. We used a paired *t*-test to determine if buffering effects differed in the two seasons. To determine the potential influence of season on the spatial patterns of forest buffering effect, we repeated the ANCOVA models described above but adding season in as an additional categorical variable.

To estimate the spatial scale over which forest edges impair the ability of forests to buffer their microclimate, we modeled buffering effect against log_4_-transformed distance to edge, treating edge distance as a continuous variable. Models were fitted separately for the three height strata. Fitted values from the model were compared to the 95% confidence intervals around fitted values in the forest interior, with edge penetration distance defined as the distance at which predicted values exceeded the upper 95% confidence interval of forest interior values [29], [30]. We used a value of 64 m as forest interior, representing the largest spatial distance over which we had field data. This value is beyond the expected penetration distance of most empirically measured forest edge effects on temperature [7], [8], [27], and beyond that expected from heat diffusion models at tropical forest edges [28]. Our chosen value is a conservative one, in that if 64 m does lie within the range of edge effects in this ecosystem, then we will under-estimate edge penetration distances.

To estimate the proportion of Atlantic forest that has experienced microclimate changes due to forest fragmentation, we applied the core area model [31], [32] which estimates the proportion of an individual fragment that is impacted by edge effects given three variables (fragment area, fragment perimeter and edge penetration distance). Using vector data on forest cover [19], we estimated the proportion of each of the 231,251 individual fragments of the Brazilian Atlantic Forest that is impacted by edge effects. Proportions were converted to area estimates by multiplying values by fragment size, summed to give a total area of forest across the biome, and then divided by total forest area to estimate the proportion of Atlantic forest. These latter two values represent the area and proportion of Atlantic forest in which the microclimate buffering effect of forests is expected to be reduced by edge effects, revealing the spatial extent of forest fragmentation on forest microclimate.

## Results

**Figure 2:**
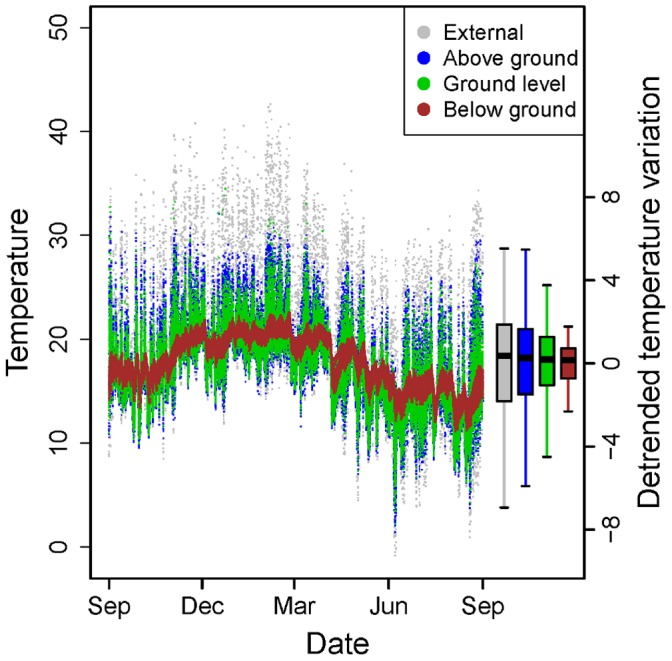
Annual variation in external air temperature (°C) and temperature inside forest at three different height strata. Points represent a single recording, taken every three hours for a 12 month period (September 1, 2009 to August 31, 2010) at 73 locations in the Brazilian Atlantic forest biome. Boxplots on the right show the residual variation in temperature after detrending. Thick lines represent the median, the box represents the upper and lower quartiles and the whiskers represent the maximum and minimum points that are no more than 1.5 times the interquartile range.

**Figure 3:**
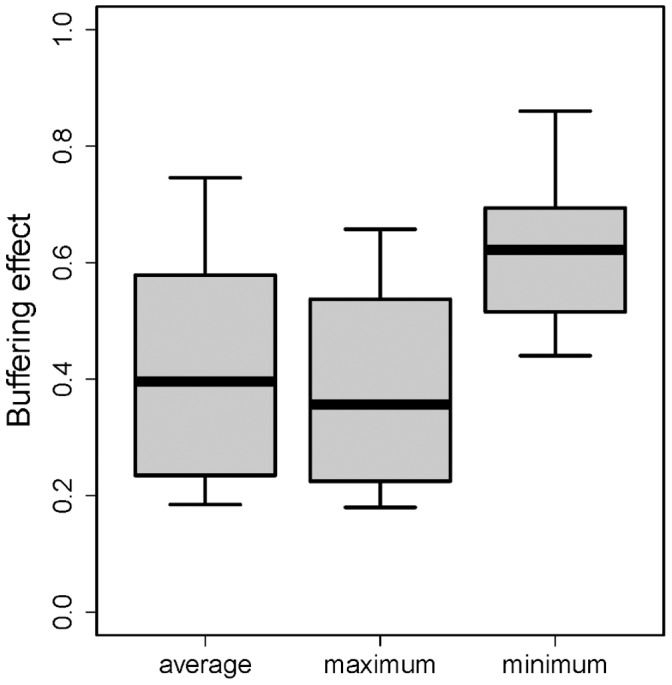
Tropical forests buffer their internal microclimate from variation in external macroclimate temperature. Buffering effect is quantified as the slope of the relationship between internal forest temperature and external temperature, with low values representing strong buffering effects. The buffering effect is strongest on average and daily maximum temperatures, but much weaker for daily minimum temperatures.

External air temperatures ranged from −0.8 to 42.6°C, but temperatures were more stable inside the forest, particularly below ground (Fig. 2). There was wide variation in the strength of buffering effects, with buffering effects on daily maximum temperatures being stronger than average buffering effects (*t* = 9.97, *P*<0.001) and daily minimum buffering effects (*t = *20.51, *P*<0.001) (Fig. 3). The slopes from linear regression models showed that for every 1°C increase in maximum temperature outside of the forest, maximum temperature inside the forest increased by just 0.38°C (SE 0.02). By contrast, for every 1°C increase in minimum temperature outside of the forest, minimum temperature inside the forest increased by 0.69°C (SE 0.01).

**Figure 4:**
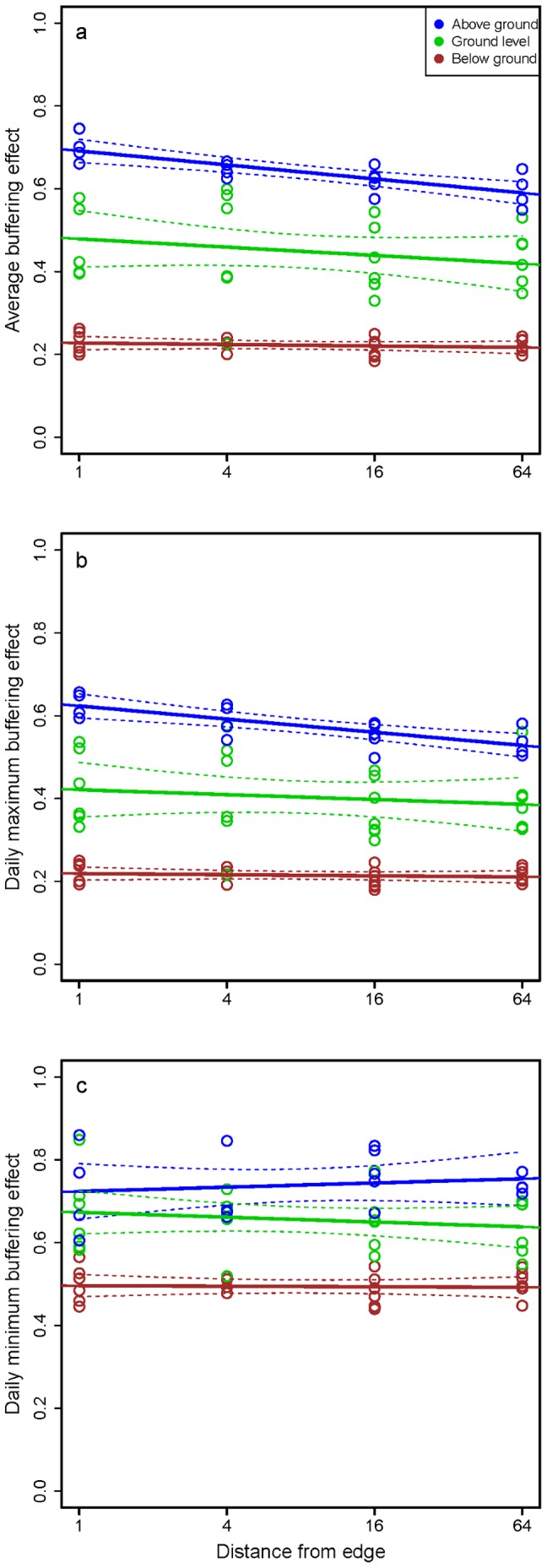
The buffering effect of tropical forest varies according to distance from forest edge and height strata. Buffering effect is quantified as the slope of the relationship between internal forest temperature and external temperature, with low values representing strong buffering effects. Results are presented separately for (a) average buffering effect, (b) buffering of daily maximum temperatures, and (c) buffering of daily minimum temperatures. The spatial structure of forests changes the magnitude of the average and daily maximum buffering effects, with the buffering effect strongest in the forest interior and decreases in strength toward the forest edge. The edge effect is strongest above ground and non-existent below ground. Distance from edge is plotted on a log_4_ scale. Thick lines represent the predicted buffering effect from models regressing buffering effect against log_4_-transformed distance from forest edges (m). Thin dashed lines represent the 95% confidence intervals around those predictions.

There were significant effects of temperature sensor location on buffering effects (Fig. 4). The average buffering effect was significantly reduced near the forest edge (*F* = 6.67, *P* = 0.012), as was the daily maximum buffering effect (*F* = 4.26, *P* = 0.043), but this pattern was not significant for the buffering effect of daily minimum temperature (*F* = 0.28, *P* = 0.580). All three buffering effects were strongest below ground and weakest above ground (average buffering effect: *F* = 373.1, *P*<0.001; daily maximum: *F* = 287, *P*<0.001; daily minimum: *F* = 160, *P*<0.001). There was, however, no significant interaction between distance to edge and height strata for any buffering effect (*F* <2.5, *P*>0.12 for all analyses).

**Figure 5:**
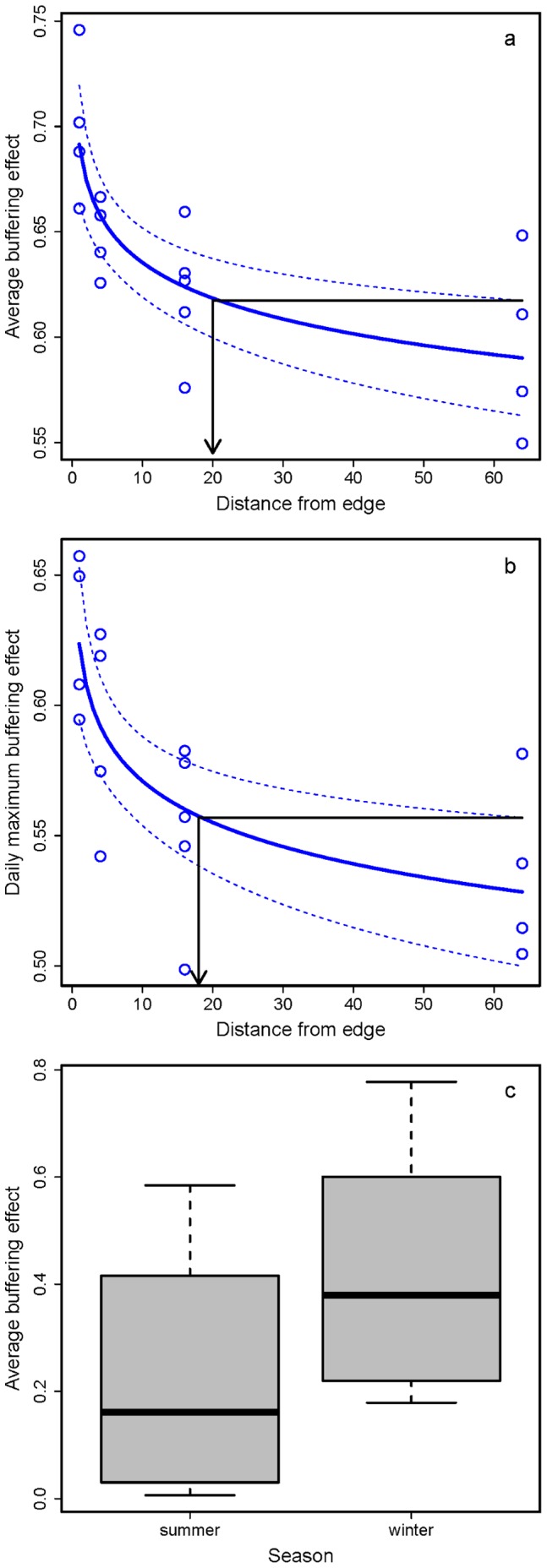
The spatial and temporal variation of temperature buffering effects in tropical forest. The effect of distance from edge on above-ground (a) average buffering effect and (b) daily maximum buffering effect, with distance from edge plotted using a linear scale. Black horizontal line and vertical arrow illustrate the calculation of edge penetration distance. (c) The average buffering effect is strongest in the summer months and weakest during winter.

The impact of distance from forest edge on the average buffering effect was detectable up to 20 m inside the forest (Fig. 5a), and 18 m inside the forest for the daily maximum buffering effect (Fig. 5b). Applying the core-area model with these edge penetration distances found that one ninth (11.6%) of the total area of the Atlantic Forest biome has experienced microclimate changes above ground.

There was a strong effect of season (Fig. 5c), with buffering effects strongest in summer and much weaker in winter (*t* = 52.2, *P*<0.001). The effects of distance from edge and height strata on average buffering effect did not significantly differ between summer and winter, with neither effect exhibiting a significant interaction term with season (season×distance from edge: *F* = 0.08, *P* = 0.78; season×height strata: *F* = 0.04, *P* = 0.85).

## Discussion

The biological relevance of small-scale edge effects depends sensitively on the spatial distribution of forest remnants [15]. Edge effects impact proportionally more forest in small fragments or in fragments with convoluted shapes, both of which have higher perimeter-to-area ratios [20]. More than 80% of forest patches within the Atlantic Forest biome are smaller than 50 ha and almost 50% of all Brazilian Atlantic forest is situated within 100 m of a forest edge [19]. Because of the heavily fragmented nature of forest in this biome and the pervasive influence of edge effects, the core-area model predicted a surprisingly large proportion of the remnant forest experiences altered microclimate conditions.

We found that edge effects on above-ground microclimate buffering effects penetrated up to 20 m inside the forest, a distance slightly lower than that reported in other microclimate studies at forest edges [4], 5,7,8,33. By contrast, we did not detect any significant edge effect on microclimate buffering effect at ground level or on below-ground soil temperature. Our study was conducted in fragments of secondary forest with relatively short stature and denser understory compared to primary Atlantic Forest. If temperatures in a forest are vertically stratified from the canopy down to the forest floor [28], it is possible that the buffering effect may be stronger in a primary forest. However, with a more open understory, edge effects should penetrate further into primary forests than what we present here. Nonetheless, our data did not encompass the upper layers of the forest canopy, so we were unable to model the attenuation of temperatures with distance below the canopy that would be necessary to accurately predict any difference between primary and secondary forests.

Combined with the heavily fragmented nature of Atlantic forest biome, the relatively small-scale edge effect of 20 m means that more than 10% of the standing forests have experienced microclimate changes arising from deforestation and forest fragmentation. This in turn suggests that less than 90% of the 160,000 km^2^ of the extant forest represents ‘core’ conditions that are free from microclimate edge effects. The core area of forest fragments can be a more accurate predictor of species richness than total forest area [34], and Atlantic Forest species are known to respond strongly to altered environmental conditions at habitat edges [22], [35]. This suggests that the effectual reduction in forest area of this biome marginally exceeds the 86–89% reported by Ribeiro et al. [19]. These figures are also likely to be a conservative estimate, as edge effects might have larger magnitudes and spatial extents in highly deforested landscapes [17], [36], [37]. Our study was conducted in a landscape with nearly 50% forest cover and where patches were separated by an average distance of just 52 m [23]. Patches in landscapes with this amount of forest cover are typically either physically or functionally connected [38], whereas connectivity is dramatically reduced in more heavily deforested landscapes. For example, in a nearby landscape of the same total size but with just 10% forest cover remaining, the average distance among patches was double what we observed in our study landscape [23]. Thus, it is possible that our results from a region of Atlantic Forest with relatively high forest cover are detecting a relatively weak influence of edge effects and that patches embedded in more heavily deforested landscapes could experience stronger edge effects. If this is true, the effectual reduction of forest area may be larger than what we report here.

We also detected significant seasonal variation in the buffering ability of forests, with the buffering effect being larger in summer than in winter. This arises because the basic ecological and physical processes that modify temperatures within a forest serve only to decrease temperature, but never to increase it. For example, the ecological process of evapotranspiration from leaves, and the physical provision of shade from the canopy, both decrease temperatures within the forest. Thus when temperatures outside the forest are particularly hot, there is a lot of potential for the forest environment to cool itself, but in winter this potential is much lower. At the same time, there is potential for the forest to retain a heat store in the soil that builds up over the warmer summer months, but then gradually releases its heat through the winter. Forests and soils have a higher heat capacity than air, and this difference suggests that decreases in forest microclimate temperatures during winter are likely to lag behind the macroclimate temperatures outside the forest, leading to a reduction in the buffering effect that is detected at the temporal scale of a single day. In combination, we believe these two processes explain the increase in buffering effects observed during summer.

Forest fragmentation is just one potential mechanism that could impair the microclimate buffering effect of forests. Our estimates of the microclimate buffering potential of tropical forests are based on an empirical model rather than a mechanistic model based on processes such as thermal diffusion [28], and ignores complexities such as topography [39], soils [40] and heterogeneity in surface material properties [41]. Similarly, we did not incorporate spatial variation in canopy structure that can be caused by processes such as wind damage, disease, herbivory or anthropogenic activities. Selective logging, for example, impacts approximately 20% of the world’s standing tropical forests [42] and thins the canopy, likely reducing the temperature buffering effect of the modified forest [28]. We suggest, then, that forest management practices that aim to maintain intact forest canopies, and forest restoration strategies that aim to reduce the perimeter-to-area ratio of fragments, represent relatively simple methods that might help mitigate the impacts of forest fragmentation on microclimate.

It is not clear whether the microclimate buffering ability of forests will help protect the forest environment from external climate changes. Inevitably, microclimate temperatures within forests will increase as the external macroclimate warms up. Our data indicate that the microclimate buffering effect of forests increases linearly with increasing macroclimate temperatures, but this effect is at least partly due to the fact that changes in forest microclimate temperatures lag behind changing macroclimate temperatures due to the higher heat capacity of forests relative to air. The length of that time lag will depend heavily on the water content of soils, with wet soils having higher heat capacity than dry soils [43]. Soil moisture in tropical forests is a balance between precipitation and water extraction by trees [44], and climate change is expected to result in increased precipitation in Atlantic forests [45]. This could lead to wetter soils [45] and therefore forest microclimate temperature changes that will lag behind those of the external macroclimate. Under this scenario, we hypothesise that the relative buffering effect of forests may increase in the short term, leading to a larger difference in temperature between the within-forest microclimate and the outside-forest macroclimate. Current data indicate that the rate at which global temperatures are rising is accelerating [46] and under these deteriorating conditions, the ability of forests to buffer microclimate from external climate change may be more important than ever.
